# Interpol: An R package for preprocessing of protein sequences

**DOI:** 10.1186/1756-0381-4-16

**Published:** 2011-06-17

**Authors:** Dominik Heider, Daniel Hoffmann

**Affiliations:** 1 Department of Bioinformatics, Center for Medical Biotechnology, University of Duisburg-Essen, Universitaetsstr. 2, 45141 Essen, Germany

## Abstract

**Background:**

Most machine learning techniques currently applied in the literature need a fixed dimensionality of input data. However, this requirement is frequently violated by real input data, such as DNA and protein sequences, that often differ in length due to insertions and deletions. It is also notable that performance in classification and regression is often improved by numerical encoding of amino acids, compared to the commonly used sparse encoding.

**Results:**

The software "Interpol" encodes amino acid sequences as numerical descriptor vectors using a database of currently 532 descriptors (mainly from AAindex), and normalizes sequences to uniform length with one of five linear or non-linear interpolation algorithms. Interpol is distributed with open source as platform independent R-package. It is typically used for preprocessing of amino acid sequences for classification or regression.

**Conclusions:**

The functionality of Interpol widens the spectrum of machine learning methods that can be applied to biological sequences, and it will in many cases improve their performance in classification and regression.

## Findings

Machine learning techniques have been widely applied to biological sequences to gain insights into biological function, for instance Rost and Sander [1], Dubchak *et al*. [2], Karchin *et al*. [3] and Nielsen *et al*. [4]. Nanni and Lumini [5] have found improved performance of classifiers based on numerically encoded amino acid sequences as compared to classifiers based on the typically used standard orthonormal representation, i.e. a vector containing twenty indicator variables (one for each amino acid) for each sequence position, resulting in a matrix containing the amino acid distributions for each position in the input sequence. For numerical encoding, each amino acid (or nucleotide) of a sequence is mapped to a numerical descriptor value, such as hydropathy [6], molecular weight, or isoelectric point.

One major limitation of almost all machine learning algorithms is the fixed input dimension, making these algorithms incapable of handling data which varies in its dimension. This is unsuitable for many biological applications as there are often sequence deletions and insertions.

We have developed a preprocessing approach for machine learning that combines the use of numerical descriptor values with a normalization of sequences to a fixed length by numerical interpolation. This procedure has already been applied to coreceptor usage prediction in HIV-1 [7], functional protein classification [8,9], and HIV-1 drug resistance prediction [10] were it led to marked improvements of prediction performance. Although many machine learning algorithms are available as software, no package for the described preprocessing of amino acid sequences is available to date. We have therefore developed Interpol, a flexible and easy to use open source package for the statistical language R http://www.r-project.org/. Currently, Interpol provides encoding of amino acid sequences with 531 different numerical descriptors from the AAindex database [11] and one additional empirical descriptor. Moreover, it allows normalization of encoded sequences to a specific length with five different linear or non-linear interpolation procedures.

Interpol is included in the Comprehensive R Archive Network (CRAN) and can be directly downloaded and installed by using the following R command:

install.packages("Interpol")

In the following example, we introduce Interpol's two commands AAdescriptor and Interpol applied to a set consisting of 1351 HIV-1 V3 loop sequences from Dybowski *et al*. [7] for the prediction of coreceptor usage (see also Table 1). After loading the set of sequences, the first V3 sequence is encoded using the AAdescriptor command:

**Table 1:** Method overview

command	parameters	information
AAdescriptor	data	amino acid sequence
	descriptor (optional)	1-532; default = 151 [6]
	normalize (optional)	0: no; 1:[-1,1]; 2:[0,1]; default = 0

Interpol	data	encoded amino acid sequence
	dims	desired length
	method (optional)	default = linear, spline, periodic, natural, fmm

library(Interpol)

data(V3)                                 #load V3 data

data.new <- AAdescriptor(V3[1])          #numerically encode sequence 1

Optional parameters are the applied descriptor (default descriptor = 151, i.e. the hydropathy scale of Kyte and Doolittle [6]) and an interval normalization (default normalize = 0, i.e. no normalization). The list of available descriptors can be found in data(list).

After encoding the amino acid sequence as numerical vector, it can be normalized to a specific length for subsequent classification. In our example, the V3 sequence lengths vary between 33 and 38 amino acids due to deletions or insertions. The following commands translate the amino acid sequences into numerical sequences using the hydropathy descriptor, and then normalize the sequences to a fixed length of 35:

library (Interpol)

data (V3)                                    #load V3 data

L.norm <- 35                                 #desired length

data.new <- matrix(nrow = length(V3),

                   ncol = L.norm)

for(i in 1:length (V3)){

  #AAdescriptor encodes sequences

  #Interpol normalizes to length L.norm

  data.new[i,] <- Interpol (AAdescriptor (V3[i]),

                            dims = L.norm)

}

**Figure 1:**
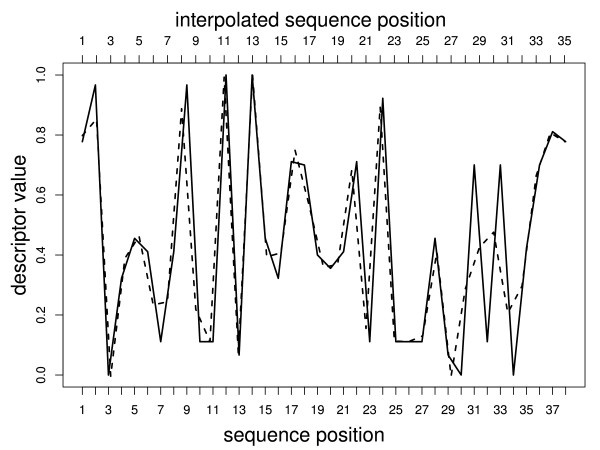
**Example: Interpolation**. A V3 loop sequence was encoded with AAdescriptor and then normalized with Interpol from length 38 to 35. Solid line: encoded sequence of length 38; dashed line: normalized sequence of length 35.

Sequence 782 in the V3 dataset has a length of 38 amino acids. In the following example the code for normalization from 38 to 35 amino acids, and for visualization of the interpolation is demonstrated (see Figure 1):

library(Interpol)

data(V3)                                    #load V3 data

sequence <- AAdescriptor(data = V3[782],    #numerically encoding

                 descriptor = 151,          #hydropathy descriptor

                 normalize = 2)             #interval normalization [0,1]

sequence.35 <- Interpol(data = sequence,    #normalize sequence

                   dims = 35,               #desired length 35

                   method="spline")         #spline interpolation

plot(sequence, type="l", ylim = c (0,1),    #plot sequence 782

            ylab="descriptor value",

            xlab="sequence position",

            lty = 1, lwd = 2)

lines(seq (1,38,(38/35)),sequence.35,     #plot normalized sequence

            lty = 2, lwd = 2)

axis(3, at = seq (1,38, 38/35),           #add axis

             labels = 1:35)

The optional parameter method can be one of linear, spline, natural, periodic or fmm (default method = linear). The linear interpolation connects two data points (*x*_0_, *y*_0_) and (*x*_1_, *y*_1_) with a straight line. The cubic spline interpolation uses piecewise cubic polynomials instead of a straight line. The spline interpolation of Forsythe [12] builds a cubic spline interpolation with the cubic passing exactly through the four points at each end of a sample. The periodic spline interpolation fulfills periodic boundary conditions, i.e. the spline curve has the same first and second derivative at its endpoints. The natural spline interpolation fulfills the natural boundary conditions.

The command help(package = Interpol) gives an overview of the Interpol package and the included methods and data. Descriptions for the AAdescriptor and the Interpol commands can be obtained by help(AAdescriptor) and help(Interpol), respectively.

In the following examples we demonstrate the use of the Interpol package for the prediction of the coreceptor usage of HIV-1 according to Dybowski *et al*. [7] based on sequences of the V3-region of the HIV-1 protein gp120. V3 is the main determinant of coreceptor usage, i.e. it determines which of the cellular coreceptors CCR5 or CXCR4 is used by HIV-1 for cell entry. Classification of V3-sequences with respect to coreceptor usage is important for therapy and prognosis. Since V3 is variable in length, many classification algorithms are not applicable. We therefore first apply AAdescriptor and Interpol to numerically encode V3-sequences and to normalize them to a fixed length. We then apply for classification random forests [13] implemented in the randomForest package, and for performance measurement the area under the receiver operating characteristics curve (AUC) implemented in the ROCR package [14] of R according to Dybowski *et al*. [7]. Note that Interpol is independent of the classification method applied, and could be also used with artificial neural networks (as in R-package neuralnet), support vector machines (as in R-package kernlab) [15], etc.

library(Interpol)

library(randomForest)

library(ROCR)

data(V3)                                                 #load V3 data

desc <- 151            #hydropathy descriptor

inter <- "linear"             #linear interpolation

L.norm <- 35                                             #desired length

classes <- c (rep (1,200), rep (0,1151))                 #class labels

data.new <- matrix (nrow = length (V3),ncol = L.norm)

for(i in 1:length(V3)){

  #AAdescriptor encodes sequences

  #Interpol normalizes to length L.norm

  data.new[i,] <- Interpol (AAdescriptor (V3[i],

                              descriptor = desc),

                              dims = L.norm,method = inter)

}

rf <- randomForest (as.factor (classes)^~^.,                #build forest

                       data = data.new)

pred <- prediction (rf$votes[,2], classes)             #prediction object

perf <- performance (pred, "auc")                         #AUC estimation

**Figure 2:**
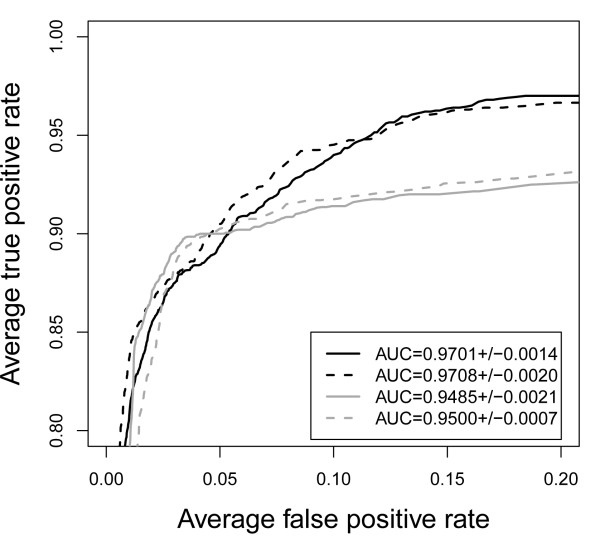
**Example: ROC curves**. Comparison of prediction performance based on different descriptors and interpolation methods implemented in Interpol and visualized with ROCR [14]. black: hydropathy (descriptor = 151); grey: net charge (descriptor = 146); solid line: linear interpolation; dashed line: spline interpolation.

Using the Interpol package, it is very easy to retrieve and compare the performance of different descriptors, e.g. hydrophobicity and net charge, and different interpolation methods (e.g. linear and spline interpolation), by just changing line desc <- 151 to desc <- 146 and inter <- "linear" to inter <- "spline", respectively, in the above code (see also Figure 2). The complete list of descriptors can be found on the help pages (help (Interpol)) and in data(list). Note that the above code somewhat overestimates the true performance as it does not include the leave-one-patient-out scheme used by Dybowski *et al*. [7].

There are several potential limitations of the Interpol method for protein classification. First, normalizing to lengths of less than 50% of the original sequence length will in general lead to loss of information. Thus, we suggest to stretch short sequences to a certain length instead of squeezing longer sequences. However, stretching can also cause problems as the normalized sequence space has a higher dimension and thus classification is more prone to overfitting. A more general limitation of normalization is that in some cases the sequence length itself can carry some information. For instance, classifying sequences of huntingtin protein [16] for induction of Huntington's disease critically relies on the length of a Glutamine repeat, an information that can be partly lost in sequence normalization.

## Availability and requirements

• Project name: Interpol

• Project home page (CRAN): http://cran.r-project.org/web/packages/Interpol/

• Operating system (s): Platform independent

• Programming language: R (≥ 2.10.0)

• License: GPL (≥ 2)

• Any restrictions to use by non-academics: none

## Competing interests

The authors declare that they have no competing interests.

## Authors' contributions

DH* has implemented and tested the software, and drafted the manuscript. DH has revised the manuscript. All authors read and approved the final manuscript.
